# Neurometabolic and functional connectivity basis of prosocial behavior in early adolescence

**DOI:** 10.1038/s41598-018-38355-z

**Published:** 2019-01-24

**Authors:** Naohiro Okada, Noriaki Yahata, Daisuke Koshiyama, Kentaro Morita, Kingo Sawada, Sho Kanata, Shinya Fujikawa, Noriko Sugimoto, Rie Toriyama, Mio Masaoka, Shinsuke Koike, Tsuyoshi Araki, Yukiko Kano, Kaori Endo, Syudo Yamasaki, Shuntaro Ando, Atsushi Nishida, Mariko Hiraiwa-Hasegawa, Richard A. E. Edden, Peter B. Barker, Akira Sawa, Kiyoto Kasai

**Affiliations:** 1 Department of Neuropsychiatry, Graduate School of Medicine, The University of Tokyo, Tokyo, Japan; 2 International Research Center for Neurointelligence (WPI-IRCN), The University of Tokyo Institutes for Advanced Study (UTIAS), The University of Tokyo, Tokyo, Japan; 3 Department of Molecular Imaging and Theranostics, National Institute of Radiological Sciences, National Institutes for Quantum and Radiological Science and Technology, Chiba, Japan; 4 Department of Psychiatry, Teikyo University School of Medicine, Tokyo, Japan; 5 The University of Tokyo Institute for Diversity and Adaptation of Human Mind (UTIDAHM), The University of Tokyo, Tokyo, Japan; 6 Department of Child Psychiatry, Graduate School of Medicine, The University of Tokyo, Tokyo, Japan; 7 Department of Psychiatry and Behavioral Sciences, Tokyo Metropolitan Institute of Medical Science, Tokyo, Japan; 8 Department of Evolutionary Studies of Biosystems, School of Advanced Sciences, The Graduate University for Advanced Studies (SOKENDAI), Kanagawa, Japan; 9 Russell H. Morgan Department of Radiology and Radiological Science, The Johns Hopkins University School of Medicine, Baltimore, MD USA; 10 F. M. Kirby Center for Functional Brain Imaging, Kennedy Krieger Institute, Baltimore, MD USA; 11 Department of Psychiatry, The Johns Hopkins University School of Medicine, Baltimore, MD USA

## Abstract

Human prosocial behavior (PB) emerges in childhood and matures during adolescence. Previous task-related functional magnetic resonance imaging (fMRI) studies have reported involvement of the medial prefrontal cortex including the anterior cingulate cortex (ACC) in social cognition in adolescence. However, neurometabolic and functional connectivity (FC) basis of PB in early adolescence remains unclear. Here, we measured GABA levels in the ACC and FC in a subsample (aged 10.5–13.4 years) of a large-scale population-based cohort with MR spectroscopy (MEGA-PRESS) and resting-state fMRI. PB was negatively correlated with GABA levels in the ACC (N = 221), and positively correlated with right ACC-seeded FC with the right precentral gyrus and the bilateral middle and posterior cingulate gyrus (N = 187). Furthermore, GABA concentrations and this FC were negatively correlated, and the FC mediated the association between GABA levels and PB (N = 171). Our results from a minimally biased, large-scale sample provide new insights into the neurometabolic and neurofunctional correlates of prosocial development during early adolescence.

## Introduction

Prosocial behavior (PB), defined as “voluntary behavior intended to benefit another”, is an important psychological factor in humans^1^. As a social process, PB facilitates complex social interactions with peers. As a psychological process, PB decreases the negative influence of stressors on emotional state^2^ and strengthens positive affect in daily life^3^, leading to increased psychological well-being^4^. Young children typically behave in a self-centered manner^5^. PB emerges in middle childhood in parallel with increases in needs-oriented prosocial reasoning^5,6^. PB further develops in late childhood influenced by an individual’s social environment including family, peers, school, and community, along with increased empathy-oriented reasoning^6,7^. Subsequently, there is a surge in the development of PB during early adolescence^8^, which is a crucial period of transition from childhood to adulthood, both physically and psychologically^9^. This is at least partly because adolescents construct more complex relationships with others and focus more on others’ acceptance and rejection^10,11^. After early adolescence, overall levels of PB decline throughout the main part of adolescence, followed by a subsequent slight rebound in early adulthood^12^. In addition, very early adolescents have equal levels of PB to different types of peers, while middle and late adolescents have differentiated PB based on the types of partners (the highest PB is towards friends and the lowest is towards anonymous partners)^13^. Thus, it would be beneficial to focus on basic or undifferentiated PB in very early adolescence, during which the peak of prosocial development is found.

Previous studies using task-related functional magnetic resonance imaging (MRI) have reported that the medial prefrontal cortex (mPFC) and the anterior cingulate cortex (ACC) are involved in the development of social cognition during adolescence^10,14^. Greater activation of the mPFC in adolescents than in adults has been reported during an animation-based mentalizing task^15^, an intentional causality comprehension task^16^, and an irony comprehension task^17^. Moreover, ACC activation during face-processing tasks increases from childhood to adolescence, and then decreases from adolescence to adulthood^18^. Such reports suggest that the mPFC, including the ACC, is a core brain region underlying the adolescent maturation of social cognition. In addition, especially the rostral ACC is responsible for social emotion^19^ and self-reference^20^ in adolescence. Therefore, it is reasonable to focus on this region and its networks to explore the neurobiological substrates underlying the development of sociality and prosociality during adolescence.

Dramatic and dynamic brain development occurs during adolescence. At a neural level, the brain undergoes extensive synaptic pruning and myelination^21^, which are both crucial for typical brain maturation. However, it is difficult to verify these neural micro-level changes *in vivo*. While macro-level developmental changes in the brain, such as cortical thinning^22^ and white matter volume increases^23^, have previously been described with structural MRI techniques, there remains little information regarding the development of neurometabolism and the functional connectome. These metabolic and functional maturation processes in adolescent brains may influence the development of prosociality, a human-specific higher-order social function^24^. However, to our knowledge, little is known about the detailed mechanisms underlying these processes.

Gamma-aminobutyric acid (GABA) is the main inhibitory neurotransmitter in the brain and GABAergic dysfunction is implicated in many psychiatric symptoms^25,26^. Magnetic resonance spectroscopy (MRS) provides a non-invasive method to measure neurotransmitters such as GABA *in vivo* in an individual’s brain^27,28^. The recent development of the MEGA-PRESS technique has overcome prior difficulties encountered in measuring GABA, such as the spectral overlap between the main GABA peaks and peaks of other neurometabolites^27,28^. Previous studies using this technique have reported lower-than-normal GABA concentrations in children with autism spectrum disorders (ASD)^29^ and attention-deficit hyperactivity disorder (ADHD)^30^. In addition, GABA levels in the ACC are negatively correlated with autistic severity in early adolescents with ASD^31^ and with impulsivity in healthy adolescents^32^. We thus hypothesize that the GABA levels in the ACC are involved in social development in adolescence. Moreover, ACC GABA levels increase with age from early adolescence to young adulthood^32^, while PB reaches at the peak in early adolescence and decreased throughout the rest part of adolescence^12^. We thus also hypothesize that the ACC GABA levels and PB are negatively associated in adolescence. However, to our knowledge, the relationship between GABA levels and PB in healthy adolescents remains to be investigated.

Resting-state functional MRI (rsfMRI) measures spontaneous blood oxygen level dependent (BOLD) signals. Therefore, it provides valuable information about brain functional connectivity (FC) independent of behavioral performance. Recently, FC has been investigated in children and adolescents. Resting-state functional networks are under genetic control in early adolescence^33^, during which they transform from anatomically-localized networks observed in children to more widely distributed networks observed in young adults^34^. Neuropsychiatric disorders in children have been investigated using rsfMRI^35^. Moreover, a recent large-scale population-based study employed rsfMRI to observe the developmental trajectory of FC in children^36^. Therefore, rsfMRI is a useful technique to elucidate neurofunctional substrates of adolescent development. In addition, ACC FC is involved in adolescent social emotion^19^ and social exclusion^37^ and is also altered in adolescents with autistic traits^38^ and ADHD^39^, which may be related to decreased prosociality^40,41^. We thus hypothesize that the functional networks of the ACC are involved in the social development in adolescence. However, to our knowledge, the association between FC and PB in adolescence remains to be uncovered.

Observation of the associations between GABA levels and FC is meaningful. GABAergic cells may modulate the BOLD signal via the inhibitory synaptic activity within excitation-inhibition microcircuits^42,43^. While only a few analyses combined GABA-MRS and rsfMRI, some studies have examined the direct association between GABA level and FC^43–46^, and most of these have revealed a negative correlation between the two^43,45,46^. These studies were limited by their small sample size. In addition, their target populations were not adolescents and their MRS regions of interest were not ACC. Despite these differences, we hypothesized a negative relationship between ACC GABA levels and ACC-seeded FC, which may affect PB.

In this context, we conducted a large-scale study focusing on PB in very early adolescence, using both GABA-MRS (MEGA-PRESS) and rsfMRI techniques. We measured GABA levels and FC in the ACC in a subsample (N = 271; 129 girls and 142 boys aged 10.5–13.4 years) of a large-scale population-based cohort. We investigated the association between PB and GABA levels in the ACC, as well as the association between PB and ACC-seeded FC. Moreover, we examined the relationship between GABA levels in the ACC and ACC-seeded FC, as well as the mediation effect of ACC-seeded FC on the association between GABA levels and PB. The strength of our study design is that participants were recruited from an ongoing population-based cohort study involving 3,171 early adolescents (Tokyo TEEN Cohort: TTC, http://ttcp.umin.jp/). This kind of study has recently been termed “population neuroscience”^47^, and may minimize the selection bias of laboratory-based neuroimaging studies.

## Results

Prior to the main analysis, we examined whether any difference in PB scale determined with the Strengths and Difficulties Questionnaire (SDQ) exists between the participants and non-participants of the pn-TTC study. The means and standard deviations were as follows: 6.8 ± 2.1 for the participants (data were missing for one); 6.7 ± 2.0 for the non-participants (data were missing for 10). We found no significant differences in SDQ PB scores between the two groups (*p* = 0.68).

### Effects of GABA on PB

**Figure 1:**
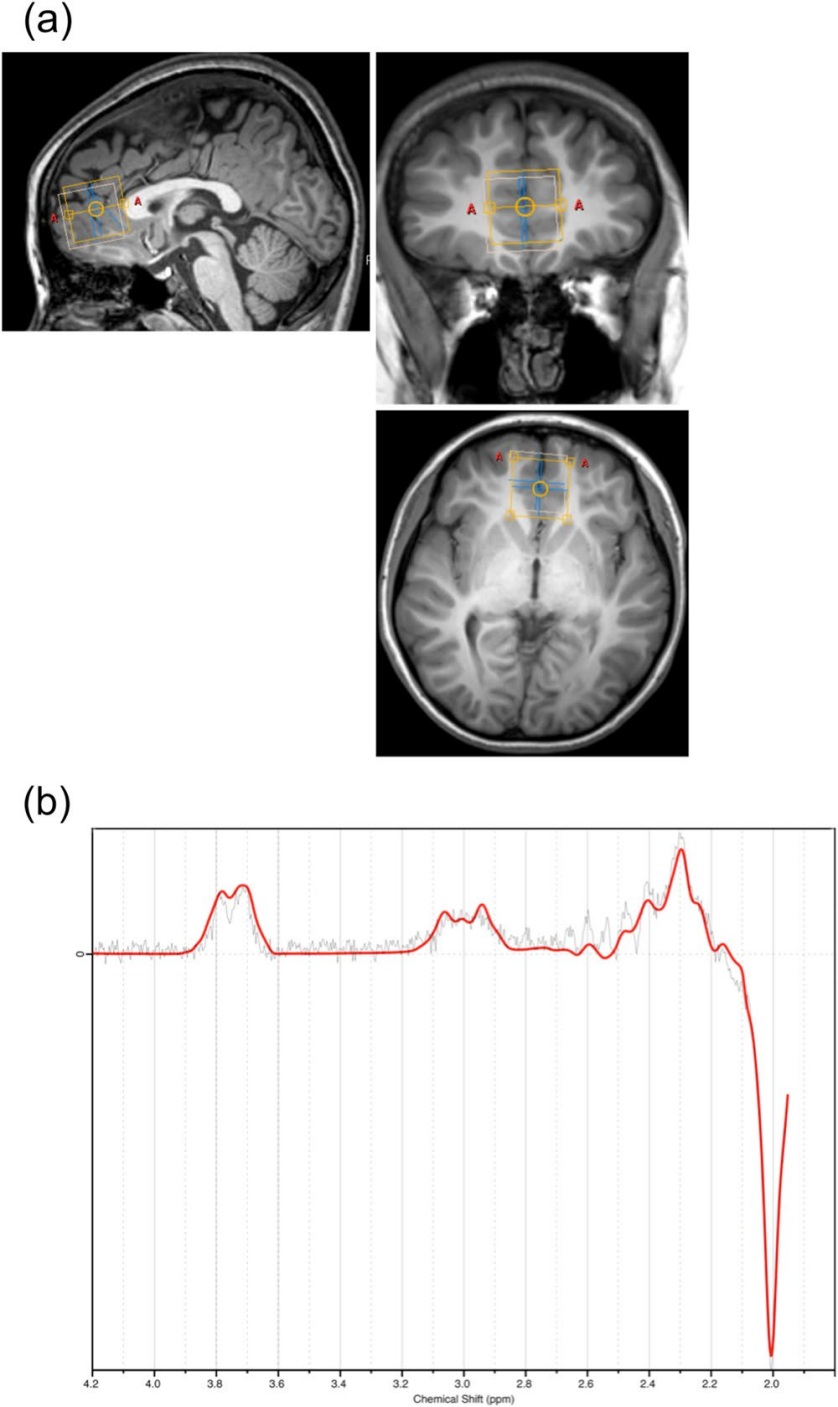
MR spectroscopy measurements. **(a)** A voxel of interest (VOI) for ^1^H MR spectroscopy was placed at the anterior cingulate cortex (ACC) in 3D-T1 anatomical images (left, sagittal plane; upper right, coronal plane; lower right, axial plane). **(b)** MEGA-editing was achieved with 15-ms Gaussian editing pulses applied at 1.90 ppm (ON) and 7.46 ppm (OFF) in alternate spectral lines. Representative actual spectra (the difference between the edit ON and OFF data) (gray line) and their LCModel fits (red line) are displayed, where the edited GABA peak (~3 ppm) is clearly detectable.

**Figure 2:**
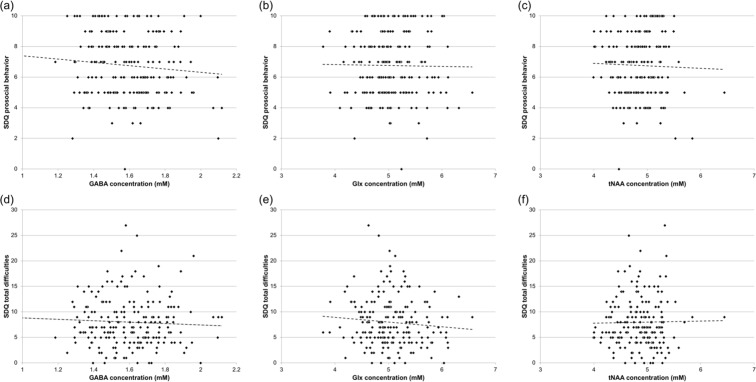
Effects of neurometabolites on Strengths and Difficulties Questionnaire (SDQ) scores. **(a)** Associations between GABA concentrations in the anterior cingulate cortex (ACC) and SDQ prosocial behavior (PB) scores. **(b)** Associations between Glx (the sum of glutamate and glutamine) concentrations in the ACC and SDQ PB scores. **(c)** Associations between tNAA (the sum of NAA and NAAG) concentrations in the ACC and SDQ PB scores. **(d)** Associations between GABA concentrations in the ACC and SDQ total difficulties (TD) scores. **(e)** Associations between Glx concentrations in the ACC and SDQ TD scores. **(f)** Associations between tNAA concentrations in the ACC and SDQ TD scores.

We acquired MRS data in the ACC with the MEGA-PRESS technique (Fig. 1a)^27,28^. From this data, we calculated GABA concentrations, concentrations of the sum of glutamate (Glu) and glutamine (Gln) (abbreviated as Glx), and concentrations of total N-acetylaspartate (tNAA), the sum of NAA and N-acetylaspartylglutamate (NAAG), by using the LCModel software (Fig. 1b). To explore the effect of GABA on prosociality, we examined associations between GABA concentrations and SDQ PB scores. We observed a significant negative correlation between GABA concentrations and SDQ PB scores (*ρ* = −0.15, *p* = 0.027) (Fig. 2a). Moreover, to explore the effect of other metabolites on prosociality, we examined associations between Glx concentrations and SDQ PB scores and between tNAA concentrations and SDQ PB scores. There was no significant correlation between SDQ PB scores and Glx (*ρ* = 8.0 × 10^−3^, *p* = 0.91) (Fig. 2b) or tNAA (*ρ* = 0.031, *p* = 0.64) concentrations (Fig. 2c). Subsequently, to investigate the effect of GABA on prosociality after the exclusion of the potential impact of other metabolites, we performed a multiple regression analysis, including GABA, Glx, and tNAA concentrations. Levels of GABA (standardized beta coefficient = −0.18, *p = *0.011), but not Glx (standardized beta coefficient = 0.044, *p* = 0.56) or tNAA (standardized beta coefficient = −0.065, *p* = 0.39), had significant effects on the SDQ PB scores. Furthermore, to explore the effect of GABA on psychological difficulties, we examined associations between GABA concentrations and SDQ total difficulties (TD) scores. We observed no significant correlation between GABA levels and SDQ TD scores (*ρ* = 4.7 × 10^−3^, *p* = 0.94) (Fig. 2d). Subsequently, we sought to determine the effect of GABA on prosociality after excluding the effect of psychological difficulties. Thus, using a multiple regression model, we calculated regression coefficients for GABA concentrations and SDQ PB scores, adjusted for SDQ TD scores. GABA levels were significantly correlated with SDQ PB scores (standardized beta coefficient = −0.16, *p* = 0.013), even when adjusted for SDQ TD scores. As additional analyses, we also tested associations between Glx concentrations and SDQ TD scores, and between tNAA concentrations and SDQ TD scores. There were no significant correlations between SDQ TD scores and Glx (*ρ* = −0.10, *p* = 0.13) (Fig. 2e) or tNAA (*ρ* = −0.055, *p* = 0.42) concentrations (Fig. 2f).

### Effects of FC on PB

**Figure 3:**
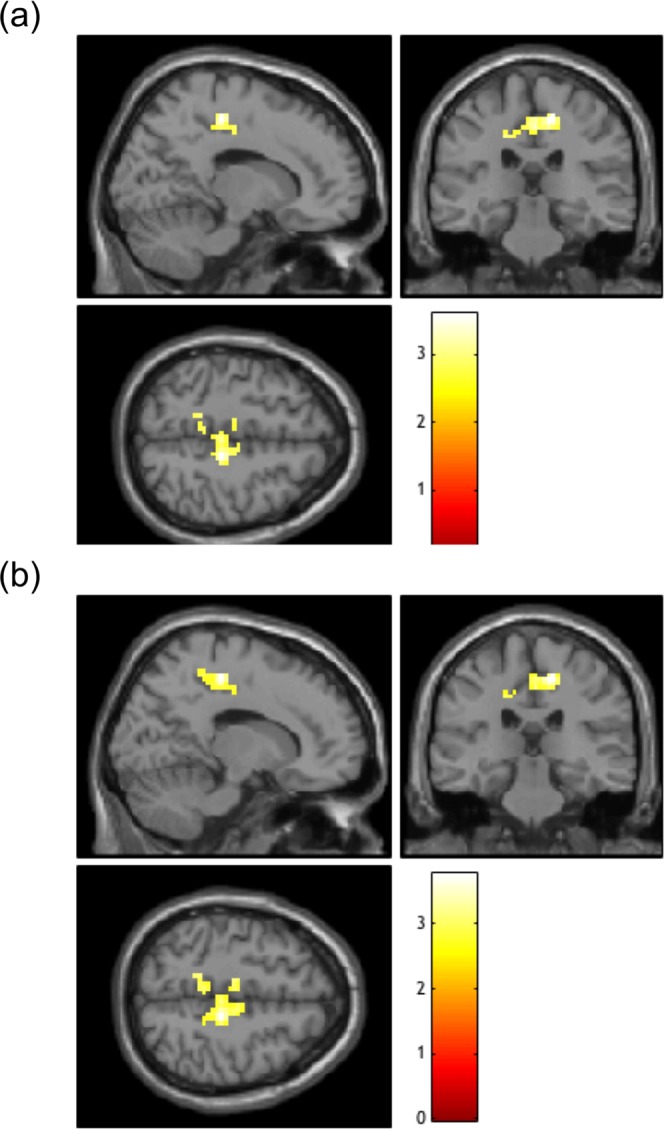
Effects of functional connectivity (FC) on prosocial behavior (PB). Brain regions in which FC to the right anterior cingulate cortex (ACC) was significantly positively correlated with Strengths and Difficulties Questionnaire (SDQ) PB scores are shown. **(a)** Brain regions showing a significant correlation without covariates. **(b)** Brain regions showing a significant correlation when controlling for SDQ total difficulties (TD) scores.

rsfMRI data were preprocessed with the Data Processing Assistant for Resting-State fMRI (DPARSF) software to create FC maps seeded in each of the bilateral ACCs. To identify brain regions in which FC with the ACC was significantly correlated with prosociality, we examined associations between seed-based FC (seeded in the left and right ACC) and SDQ PB scores. We observed that SDQ PB scores were significantly positively correlated with right ACC-seeded FC with the right precentral gyrus and the bilateral middle and posterior cingulate cortex (MCC and PCC) (peak voxel MNIxyz = [15–27 51]) (Fig. 3a and Table 1). Moreover, to identify brain regions in which FC with the ACC was significantly correlated with psychological difficulties, we examined associations between seed-based FC (seeded in the left and right ACC) and SDQ TD scores. We did not observe any significant correlation between ACC-seeded FC and SDQ TD scores. Subsequently, we sought to observe correlations between ACC-seeded FC and SDQ PB scores after controlling for the effects of psychological difficulties. Thus, we examined associations between seed-based FC (seeded in the left and right ACC) and SDQ PB scores adjusted for SDQ TD scores. We observed that SDQ PB scores were significantly positively correlated with right ACC-seeded FC with the right precentral gyrus and the bilateral MCC and PCC, even when adjusted for SDQ TD scores (peak voxel MNIxyz = [15–27 51]) (Fig. 3b and Table 2).

**Table 1:** Peak MNI coordinates for regions showing significant correlations between ACC-seeded FC and SDQ PB scores.

	Region	Peak coordinates				Cluster size	Cluster-level FWE-corrected *p*
		x	y	z	t		
**Positive correlation**							
Seed: Left ACC	None						
Seed: Right ACC	Right precentral gyrus	15	−27	51	3.58	191	0.016
	Right middle cingulate gyrus	9	−21	45	3.28		
	Left middle cingulate gyrus	−6	−18	45	3.28		
**Negative correlation**							
Seed: Left ACC	None						
Seed: Right ACC	None						

Statistical thresholds were set at an uncorrected *p* < 0.005 (voxel level) and a family-wise error (FWE)-corrected *p* < 0.05 (cluster level).

Abbreviations: ACC, anterior cingulate cortex; FC, functional connectivity; SDQ, Strengths and Difficulties Questionnaire; PB, prosocial behavior; L, left; R, right.

**Table 2:** Peak MNI coordinates for regions showing significant correlations between ACC-seeded FC and SDQ PB score, adjusted for SDQ TD scores.

	Region	Peak coordinates				Cluster size	Cluster-level FWE-corrected *p*
		x	y	z	t		
**Positive correlation**							
Seed: Left ACC	None						
Seed: Right ACC	Right precentral gyrus	15	−27	51	3.75	265	0.015
	Left middle cingulate gyrus	−6	−15	45	3.59		
	Right middle cingulate gyrus	12	−18	45	3.29		
**Negative correlation**							
Seed: Left ACC	None						
Seed: Right ACC	None						

Statistical thresholds were set at an uncorrected *p* < 0.005 (voxel level) and a family-wise error (FWE)-corrected *p* < 0.05 (cluster level).

Abbreviations: ACC, anterior cingulate cortex; FC, functional connectivity; SDQ, Strengths and Difficulties Questionnaire; PB, prosocial behavior; TD, total difficulties; L, left; R, right.

### Correlations between GABA and FC

**Figure 4:**
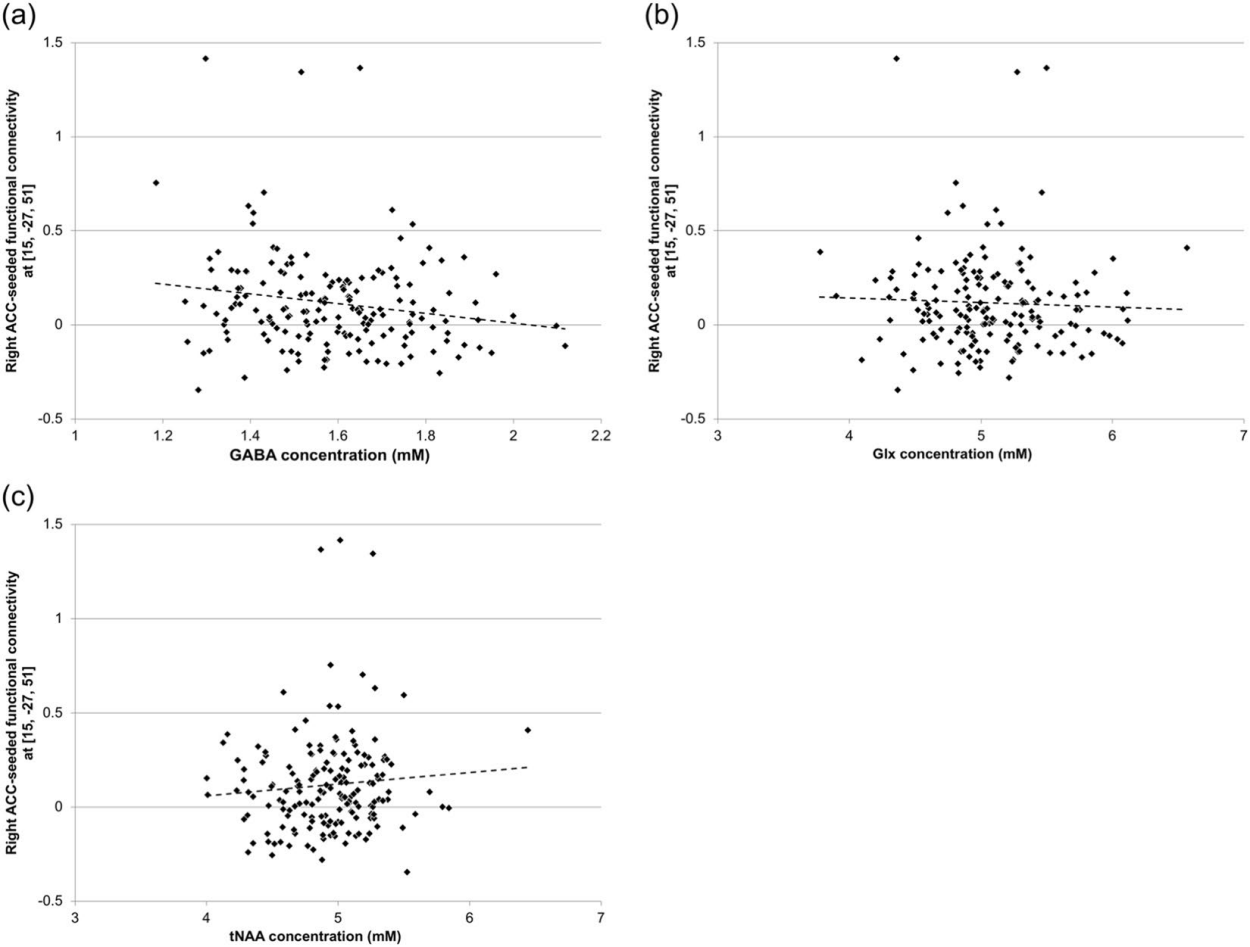
Effects of neurometabolites on functional connectivity (FC). **(a)** Associations between GABA concentration in the anterior cingulate cortex (ACC) and right ACC-seeded FC to the peak voxel (MNIxyz = [15–27 51]) in the cluster, in which right ACC-seeded FC was positively correlated with Strengths and Difficulties Questionnaire (SDQ) prosocial behavior (PB) scores. **(b)** Associations between Glx (the sum of glutamate and glutamine) concentrations in the ACC and the same FC. **(c)** Associations between tNAA (the sum of NAA and NAAG) concentrations in the ACC and the same FC.

We observed that SDQ PB scores were positively correlated with right ACC-seeded FC, with the cluster region including the right precentral gyrus and the bilateral MCC and PCC (peak voxel MNIxyz = [15–27 51]). Therefore, to identify any relationship between GABA and FC, both of which affect prosociality, we investigated correlations between GABA concentrations and the right ACC-seeded FC to the peak voxel in the cluster. We observed a significant negative correlation between GABA levels and this functional connection (*ρ* = −0.17, *p* = 0.029) (Fig. 4a). Moreover, to explore the effects of other metabolites, we examined associations of Glx and tNAA concentrations with this functional connection. We did not observe any significant correlation between the functional connection and Glx (*ρ* = −0.059, *p* = 0.44) (Fig. 4b) or tNAA (*ρ* = 0.077, *p* = 0.31) concentrations (Fig. 4c). Furthermore, to explore the effect of GABA on this functional connection after excluding the potential impact of other metabolites, we calculated regression coefficients for GABA concentrations and this functional connection, adjusted for Glx and tNAA concentrations. There was a significant correlation between GABA levels and this functional connection (standardized beta coefficient = −0.16, *p* = 0.048), even when adjusted for Glx and tNAA concentrations.

### Mediation effects of FC on the association between GABA and PB

**Figure 5:**
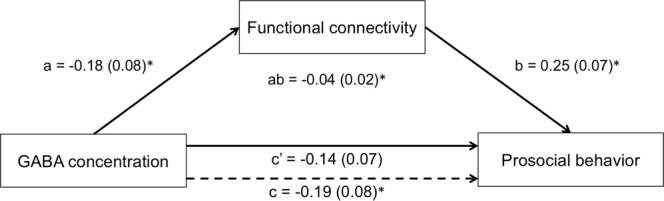
Mediation effect of functional connectivity (FC) on the association between GABA and prosocial behavior (PB). The effect of GABA concentrations in the anterior cingulate cortex (ACC) on PB through right ACC-seeded FC to the peak voxel (MNIxyz = [15–27 51]) in the cluster, in which right ACC-seeded FC was positively correlated with Strengths and Difficulties Questionnaire (SDQ) PB scores, is illustrated. Standardized path coefficients (standard error) are shown for each path. *p < 0.05.

To explore the effects of GABA and ACC-seeded FC on PB in more detail, we conducted a mediation analysis to assess whether ACC-seeded FC to the peak voxel in the above cluster mediates the effect of GABA concentrations on PB. The mediation model was found to account for significant variance in PB (*R*^2^ = 0.094, *F* = 8.7, *p* = 0.0003). The total effect of GABA concentrations on PB was significant (standardized beta coefficient = −0.19, *p* = 0.014), while the direct effect was found to be marginally significant (standardized beta coefficient = −0.14, *p* = 0.057). Moreover, there was a significant negative indirect effect of GABA concentrations on PB through FC (standardized beta coefficient = −0.044, 95% confidence interval = [−0.10, −0.012]). The results of the mediation analysis are summarized in Fig. 5.

### Age effects on PB, GABA, and FC

**Figure 6:**
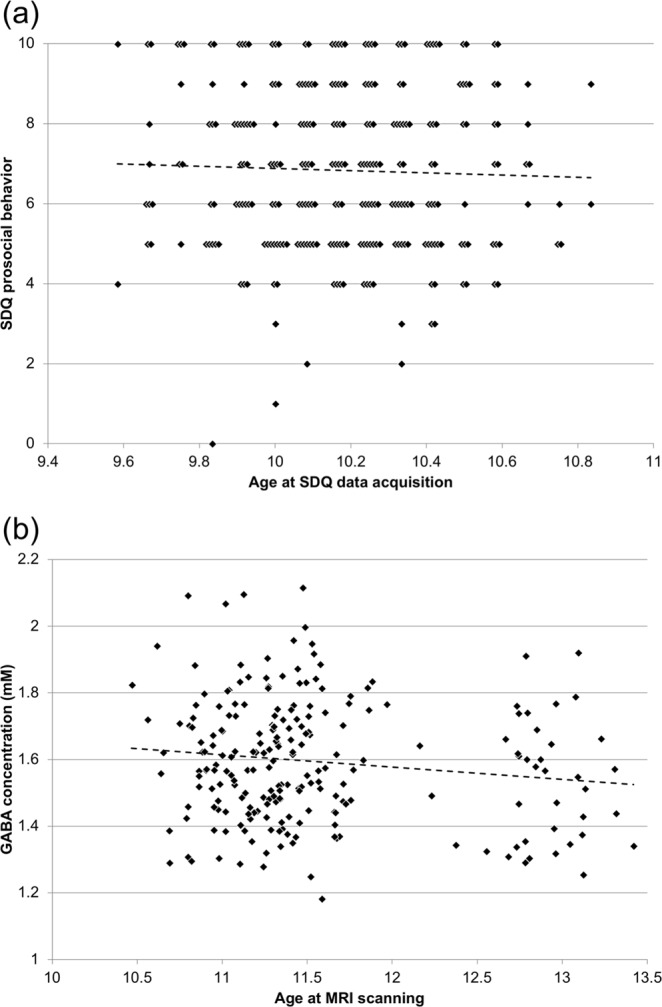
Effects of age. **(a)** Association between age at acquisition of data on the Strengths and Difficulties Questionnaire (SDQ) and SDQ prosocial behavior (PB) scores in all participants (data for one participant missing). Multiple points that are originally on the same coordinate are shown separately for display purpose. **(b)** Association between age at MRI scanning and GABA concentrations in the anterior cingulate cortex (ACC).

To verify the existence of acute developmental changes in prosociality, GABA levels, and FC, we sought to explore the effect of age on these factors within the current cohort sample. We examined associations between age at SDQ data acquisition and SDQ PB scale in all participants (data for one participant were missing). There was no significant correlation between age and SDQ PB scale (*ρ* = −0.044, *p* = 0.47) (Fig. 6a). Subsequently, we investigated the correlation between age at MRI scanning and GABA levels. We observed no significant correlation between age and GABA concentrations (*ρ* = −0.079, *p* = 0.24) (Fig. 6b). We also examined whether seed-based FC (seeded in the left and right ACC) was correlated with age at MRI scanning, and found no brain region in which FC with the ACC was significantly correlated with age. In addition, for a contrast analysis, we examined associations between age at SDQ data acquisition and SDQ PB scale in the non-participants of the pn-TTC study (data were missing for 14 individuals). We found no significant correlation between age and SDQ PB scale (*ρ* = −0.011, *p* = 0.58).

### Supplementary analyses

We also conducted supplementary analyses of associations between PB and psychological difficulties (Supplementary Fig. 1), of associations between GABA and other metabolites (Supplementary Fig. 2), and of sex differences in PB, GABA, and FC (Supplementary Fig. 3). Detailed results are provided in Supplementary Results.

## Discussion

Our study revealed that PB is negatively correlated with GABA levels in the ACC and positively correlated with right ACC-seeded FC with the right precentral gyrus and the bilateral MCC and PCC. The effects of GABA and FC on PB remained significant when controlling for the effects of psychological difficulties. Moreover, we observed a negative correlation between GABA concentrations and this FC, and a mediation effect of the FC on the association between GABA levels and PB. The effect of GABA on PB and FC remained significant when controlling for effects of other neurometabolites. Taken together, our results provide new insights into the neurometabolic and neurofunctional correlates of prosocial development during early adolescence.

We demonstrated a significant negative correlation between PB and GABA levels in the ACC (Fig. 2a). To our knowledge, our results are the first to characterize the negative relationship between prosociality and GABA levels in early adolescents. Prior studies using MRS have investigated GABA levels in children with ASD, in whom prosociality may be impaired^40,48^. Some reports reported lower-than-normal GABA levels in auditory and visual brain regions in children with ASD^29,49^, while another study reported unaltered GABA levels in these patients^31^. However, our results are not in accordance with such findings. We assume that differences in the results across studies can be ascribed to differences in MRS voxels of interest (VOI), scanners, sample sizes, and medication use. In rodent models, an excitatory-to-inhibitory developmental switch in GABA activity, triggered by oxytocin at delivery, continues until late adolescence, and disturbances of this switch cause autistic-like features^50,51^. This does not support the hypothesis of a reduction in the total amount of GABA in ASD. Oblak *et al*.^52^ reported a decrease in GABA_A_ receptor density in the ACC in ASD, and assumed that this might result from an increase in GABA release, which would be consistent with our results. The GABA levels in the ACC explain a significant but small portion of the variability in PB (Fig. 2a), which may be affected by potential confounding factors, including sex^53^, adolescent developmental stages^54^, menstrual cycle phases (in girls)^55^, and psychopathology. Owing to such variability of GABA levels and PB, their negative correlation should be interpreted cautiously.

We also observed a significant positive correlation between PB and right ACC-seeded FC with regions including the right precentral gyrus and the bilateral MCC and PCC (Fig. 3a and Table 1). The PCC/precuneus region and the ACC/mPFC region are the most important core hubs of the default mode network (DMN), defined by their strong correlations with all regions constituting the DMN^56^. FC between these two regions is highly related to social functioning, such as perceived social support^57^ and empathizing^58^. Such anterior-posterior FC is low in children^59^, increases with age^60^, and is highly developed in adults^61^. It is thus suggested that the development of ACC-PCC FC should underlie maturation processes of prosociality from childhood to adulthood. In contrast, in individuals with ASD, ACC-PCC FC decreases with age^62^ and is lower than in healthy individuals^62–64^. Anterior-posterior FC is also reduced in adolescents with conduct disorder, in whom prosociality may be impaired^65^. Collectively, we assume that the “prosociality continuum” represented neurally by the functioning of ACC-PCC FC, encompasses the entire spectrum of normal prosociality, subclinical poor prosociality, and clinically-diagnosed impairments in prosociality. To our knowledge, our results are the first to reveal an association between prosociality and FC in early adolescents.

We observed a significant negative correlation between GABA concentrations and right ACC-seeded FC to the peak voxel in the cluster, including the PCC (Fig. 4a). Several previous studies in smaller samples have reported negative correlations between GABA levels and FC^43,45^. We have successfully replicated these previous results in a large-scale study. However, it reamins unclear how local GABA reduces distant resting-state functional connectivity, while it clearly reduces local hemodynamic changes during a task^66^. Further research is required to elucidate the detailed physiological mechanisms accompanying this association.

Our mediation analysis revealed a significant indirect effect of ACC GABA concentrations on PB through anterior-posterior FC in the DMN, although the direct effect was found to be only marginally significant (Fig. 5). To our knowledge, this is the first study to reveal that a neurotransimitter influences psychological features via functional brain connectivity. We suggest that GABA in the ACC should decrease PB by interfering with the synchronization of hemodynamics between the anterior and posterior DMNs. This pathway may be a key treatment target for adolescent individuals with impaired prosociality. Genetic biological effects on PB increase with age from childhood to adulthood^67^. We thus assume that the revealed biological pathway may become evident with growth and that the pathway may be weaker in childhood and stronger in adulthood than in adolescence. Further studies will be required to solve this possibility.

Our data revealed no significant age associations with PB within the present study sample (Fig. 6a). In addition, we found no age associations with PB in the non-participants of the pn-TTC survey. Although prosociality does not develop immediately within a narrow age range (between the age of 9 to 11 years), it gradually matures from childhood to early adolescence^6^. However, the assessment results of prosocial development may be different across raters (parents and teachers)^68^ or cultural backgrounds^69^, while to our knowledge, neither puberty onset nor the entry into secondary education affects prosociality. Thus, our result of no age association with PB should be interpreted cautiously considering these issues.

Our data revealed no significant age associations with GABA level within the present study sample (Fig. 6b). A previous MRS study reported that ACC GABA levels were significantly lower in early adolescents than in emerging adults^32^, while few MRS studies examined GABAergic maturation during adolescence. We thus assume that during adolescence, GABA levels may increase not immediately but gradually. Our data revealed no significant age associations with ACC FC within the present study sample. Previous rsfMRI studies reported that FC between the ACC/mPFC and PCC within DMN were lower in children than in young adults^59,70^. We suggest that during adolescence, FC between ACC/mPFC and PCC develops not immediately but gradually, which leads to prosocial development. Collectively, further research will be needed to elucidate the dynamic mechanisms of adolescent prosocial development, including neurobiological processes.

The current study is subject to several limitations. First, neurometabolite levels measured with MRS include not only functioning neurotransmitters, but also pools that are not used as neurotransmitters. Therefore, we cannot evaluate the exact synaptic GABA signaling state with this technique. Second, spectra acquisition was limited to one VOI because of time constrains and a desire to avoid participants having to endure undue stress. However, it would have been ideal to extract spectra from another VOI as a control region. Third, the ACC VOI in the MRS analyses and the ACC seed region in the FC analyses were not completely co-localized but only partially overlapped for technical reasons. Fourth, it is unclear to which extent the parent-rated SDQ PB scores are related to prosociality in real life. According to a large-scale review, the inter-rater correlation on the SDQ PB scores between parents and teachers was relatively low (0.26)^71^. We at least assume that the parent-rated assessment does not cover overall prosociality in real life. Thus, teacher-rated assessment should also be ideally included. Finally, SDQ data were acquired, on average, 16 months prior to MRI scanning. Thus, because of a relatively large time gap, the reliability of our results cannot be fully guaranteed. However, age-related changes in PB, GABA levels, or FC were not observed within the current study sample (Fig. 6). Therefore, we suggest that intra-individual longitudinal changes in these factors within such a narrow age range would be small. In addition, early adolescents with high/low prosociality will follow a high/low prosociality trajectory until late adolescence^72^. Thus, the issue of time intervals is not be so problematic in our current analyses. However, to overcome this limitation, we would ideally need longitudinal data.

In conclusion, the current “population neuroscience” study with a minimally biased, large-scale sample provides new insights into the neurometabolic and neurofunctional correlates of prosocial development during early adolescence.

## Methods

### Participants

This study was conducted as part of the population-neuroscience study of the TTC (pn-TTC) study, in which 301 early adolescents were recruited from the general population. Participants of the pn-TTC study were subsampled from a larger participant group of the TTC study, and it was confirmed that the pn-TTC subsample was representative of the TTC study population. Written informed consent was obtained from each participant and the participant’s primary parent before participation. All protocols were approved by the research ethics committees of the Graduate School of Medicine and Faculty of Medicine at the University of Tokyo, Tokyo Metropolitan Institute of Medical Science, and the Graduate University for Advanced Studies (SOKENDAI). All research was performed in accordance with relevant guidelines/regulations. The detailed methods for participant recruitment are described in Supplementary Method 1.

### Image acquisition

MRI scanning was performed on a Philips Achieva 3T system (Philips Medical Systems, Best, The Netherlands) with an eight-channel receive head coil. Each participant underwent an MRI examination comprising fluid attenuated inversion recovery (FLAIR), rsfMRI, a T1 three-dimensional (3D) magnetization-prepared rapid gradient echo sequence (3D-MPRAGE), MR angiography (MRA), and MRS sequences. However, not all participants underwent all the above-mentioned sequences for various reasons, such as interruption of scanning due to participant fatigue.

The rsfMRI data were acquired with a gradient-echo echo-planar imaging (EPI) sequence with the following parameters: repetition time (TR)/echo time (TE), 2500 ms/30 ms; flip angle, 80°; acquisition matrix, 64 × 59; field of view (FOV), 212 mm × 199 mm × 159 mm; voxel size, 3.31 mm × 3.37 mm × 3.20 mm; slice thickness, 3.20 mm; slice gap, 0.8 mm. Each brain volume consisted of 40 axial slices, and each functional run contained 250 image volumes preceded by four dummy volumes, resulting in a total scan duration of 10 min 40 sec. Participants were asked to stay awake, not to focus their thoughts on anything as far as possible, and to keep their eyes on a fixation point at the center of the screen during rsfMRI scanning.

^1^H MR spectra were acquired from a 30 × 30 × 30 mm^3^ VOI positioned in the ACC with the MEGA-PRESS method^27,28^. The VOI in the ACC was positioned anterior and close to the tip of the genu of the corpus callosum and centered on the interhemispheric fissure (Fig. 1a). We used a T1 3D-MPRAGE sequence with an isotropic voxel resolution of 1 mm^3^ for MRS voxel placement. Spectral data were acquired with the following parameters: TR/TE, 2000 ms/68 ms; dynamic scans, 160; sample points, 2048; bandwidth, 2000 Hz; phase cycle, 16. The acquisition time was approximately 10 min 42 sec. MEGA-editing was achieved with 15-ms Gaussian editing pulses applied at 1.90 ppm (ON) and 7.46 ppm (OFF) in alternate spectral lines. Water suppression was achieved with the multiply optimized insensitive suppression train (MOIST) water suppression technique (for Philips scanners).

### Image preprocessing

rsfMRI data preprocessing was performed with DPARSF software version 2.1^73^, which is a MATLAB (MathWorks, Natick, MA) toolbox. Imaging data were adjusted for temporal shifts in acquisition, spatially realigned to the middle slice, nonlinearly normalized to the EPI MNI template (resulting in an isotropic voxel size of 3 mm^3^), smoothed with a full-width at half-maximum (FWHM) kernel of 4 mm, and bandpass filtered (0.01–0.1 Hz). The following nuisance signals were regressed out of the time course of each voxel: head motion time series estimated with the Friston 24-parameter model (head motion parameters from realigned data including 6 head motion parameters, 6 head motion parameters one time point before, and the 12 corresponding squared items)^74^; head motion scrubbing regressors (time points with more than 0.5 mm of framewise displacement and 1 back and 2 forward neighboring points were modeled as a regressor)^75^; and white matter and cerebrospinal fluid signals. Finally, time course data were processed for each of the 90 brain regions (excluding the cerebellum) defined by the automated anatomical labeling (AAL) atlas^76^ by averaging over voxels within each region. Intra-regional correlation coefficients were calculated between every pair of an AAL region and a voxel, and connectivity maps were created. Correlation coefficients representing FC were converted to z-scores with Fisher’s transformation. The head motion parameters of all participants during rsfMRI scanning were recorded, and data were excluded from further analyses if the maximum translation exceeded 3 mm or if the maximum rotation exceeded 3 degrees in any direction^77,78^. In the following analyses, we used FC maps seeded in the left and right ACC. By using the AAL atlas, the ACC regions were delimited by the paracingulate sulcus rostrally and by the corpus callosum caudally, and were dissociated from the median cingulate and medial superior frontal regions^76^.

All MRS data were quantified with the LCModel version 6.3^79^, a frequency domain spectral fitting program (Fig. 1b). We measured signals of GABA as a metabolite of interest in the current study. We also used Glx and tNAA as reference metabolites. The LCModel calculates neurometabolite levels by referencing to the unsuppressed water peak. Water scaling and fitting were automatically conducted. Cramer-Rao Lower Bounds (CRLB) was used for the expression of uncertainties in quantifying metabolite levels. Only metabolite spectra with LCModel-estimated uncertainty of <10% standard deviations (SDs) were included in this study, to reject low-quality spectra.

### Psychological evaluation

In the current study, we used PB subscale and TD scale of the SDQ^80^ as a variable of interest and a reference variable, respectively. The detailed explanations for the SDQ are described in Supplementary Method 2.

### Subject selection

We had four neuroimaging samples: all participants (N = 271), the GABA-MRS analysis sample (N = 221), the rsfMRI analysis sample (N = 187), and the combination analysis sample (N = 171). The detailed procedures for subject selection are described in Supplementary Method 3. Basic demographic characteristics did not differ between the four neuroimaging samples (Table 3).

**Table 3:** Participant characteristics.

	Overall							Girls							Boys						
	N	Age		SDQ PB		SDQ TD		N	Age		SDQ PB		SDQ TD		N	Age		SDQ PB		SDQ TD	
		M	SD	M	SD	M	SD		M	SD	M	SD	M	SD		M	SD	M	SD	M	SD
All participants	271	11.5	0.7	6.8^a^	2.1^a^	8.0^a^	4.7^a^	129	11.5	0.6	7.0	2.0	7.7	4.4	142	11.6	0.7	6.6^a^	2.2^a^	8.2^a^	5.0^a^
GABA-MRS analysis sample	221	11.5	0.7	6.8	2.1	7.9	4.8	106	11.5	0.6	6.9	2.0	7.7	4.4	115	11.6	0.7	6.6	2.2	8.1	5.1
rsfMRI analysis sample	187	11.6	0.7	6.8	2.2	7.8	4.8	92	11.6	0.7	7.1	2.0	7.7	4.7	95	11.7	0.8	6.6	2.3	8.0	4.9
Combination analysis sample	171	11.6	0.7	6.8	2.2	7.7	4.8	83	11.6	0.7	7.1	2.0	7.6	4.7	88	11.7	0.8	6.6	2.3	7.8	4.9

^a^SDQ Data were missing for one individual.

Abbreviation: SDQ, Strengths and Difficulties Questionnaire; PB, prosocial behavior; TD, total difficulties; M, mean; SD, standard deviation; MRS, Magnetic resonance spectroscopy; rsfMRI, resting-state functional MRI.

### Statistical analyses

All statistical analyses except for second-level imaging analyses were conducted with SPSS version 24.0.0 (IBM, New York, NY, USA). Second-level analyses of seed-based FC maps were performed in SPM12 (Wellcome Department of Cognitive Neurology, London, UK). We set the type-I error rate (*p* value) at 0.05. For second-level analysis of FC maps, the statistical threshold was set at an uncorrected *p* < 0.005 at voxel level and at a whole-brain family-wise error (FWE)-corrected *p* < 0.05 at cluster level^81,82^. All assumptions of statistical tests were met.

Prior to the main analysis, we examined whether any difference in SDQ PB scale exists between the participants (N = 301) and non-participants (N = 2,870) of the pn-TTC study, among the participants in the TTC survey (the whole cohort). This analysis was performed using Mann-Whitney *U*-tests.

First, to explore the effect of GABA on prosociality, we examined associations between SDQ PB scores and GABA concentrations with Spearman’s rank tests. Moreover, to explore the effects of other metabolites on prosociality, we examined associations between SDQ PB scores and Glx concentrations and those between SDQ PB scores and tNAA concentrations with Spearman’s rank tests. Subsequently, we sought to determine the effect of GABA on prosociality after excluding the potential impact of other metabolites. Thus, using a multiple regression model, we calculated regression coefficients for GABA concentrations and SDQ PB scores, adjusted for Glx and tNAA concentrations. Furthermore, to explore the effect of GABA on psychological difficulties, we examined associations between SDQ TD scores and GABA concentrations with Spearman’s rank tests. We next sought to determine the effect of GABA on prosociality after excluding the effects of psychological difficulties. Thus, using a multiple regression model, we calculated regression coefficients for GABA concentrations and SDQ PB scores, adjusted for SDQ TD scores. As additional analyses, we also tested associations between SDQ TD scores and Glx concentrations, and between SDQ TD scores and tNAA concentrations with Spearman’s rank tests.

Second, to identify brain regions in which FC with the ACC was significantly correlated with prosociality, we examined associations between SDQ PB scores and seed-based FC (seeded in the left and right ACC) with second-level linear regression analyses. Moreover, to identify brain regions in which FC with the ACC was significantly correlated with psychological difficulties, we examined associations between SDQ TD scores and seed-based FC (seeded in the left and right ACC) with second-level linear regression analyses. Subsequently, we sought to investigate any correlation between ACC-seeded FC and SDQ PB scores after excluding the effects of psychological difficulties. Thus, we examined associations between SDQ PB scores and seed-based FC (seeded in the left and right ACC) adjusted for SDQ TD scores, with second-level linear regression analyses. All these analyses were conducted in SPM12.

Third, we sought to identify the relationship between GABA and FC, both of which might affect prosociality. Therefore, if we observed any brain regions in which FC with the ACC was correlated with SDQ PB scores in the preceding analysis, we examined correlations between GABA concentrations and the ACC-seeded FC to the peak voxel in a cluster with Spearman’s rank tests. Moreover, to explore the effects of other metabolites on this functional connection, we examined associations between Glx and tNAA concentrations and this functional connection with Spearman’s rank tests. Furthermore, we sought to elucidate the effect of GABA on this functional connection after excluding the potential impact of other metabolites. Thus, using a multiple regression model, we calculated regression coefficients for GABA concentrations and this functional connection, adjusted for Glx and tNAA concentrations.

Fourth, to explore the detailed mechanistic effect of GABA and ACC-seeded FC on PB, we conducted a mediation analysis to assess whether ACC-seeded FC mediates the effect of GABA concentrations on PB. The circuits and physiology are generally regarded as an endophenotype mediating small level biological systems (genes, cells, and molecules) and psycho-behavioral characteristics. Specifically, if we observed any brain regions in which ACC-seeded FC was significantly correlated with SDQ PB scores in the preceding analysis, we assessed whether ACC-seeded FC to the peak voxel in a significant cluster mediated the effect of GABA concentrations on SDQ PB scores. This mediation analysis was performed by using the PROCESS plugin for SPSS^83^ with 5000 bootstrapped samples.

Fifth, to verify whether acute developmental changes in prosociality, GABA levels, or ACC-seeded FC exist, we sought to explore age effects on these factors within the current sample. We examined associations between age at SDQ data acquisition and SDQ PB scale, as well as between age at MRI scanning and GABA levels, with Spearman’s rank tests. We also checked whether seed-based FC (seeded in the left and right ACC) was correlated with age at MRI scanning with second-level linear regression analyses in SPM12. In addition, for a contrast analysis, we checked the age effects on prosociality in the non-participants of the pn-TTC study (N = 2,870). We examined associations between age at SDQ data acquisition and SDQ PB scale with Spearman’s rank tests.

We also conducted the following supplementary analyses. The detailed methods for supplementary analyses are described in Supplementary Method 4.

Finally, we conducted power analyses to ensure that the current study was sufficiently powered. A priori power analyses (*α* = 0.05, 1−*β* = 0.80, two-sided tests) using G*Power 3.1.9.2^84^ revealed that the medium effect sizes for a correlational analysis (*r* = 0.30) and those for a multiple regression analysis (*f*^2^ = 0.15)^85^ could be detected in a sample with a size of 82 and 55, respectively. The sample sizes in all analyses in this study were adequate.

## Supplementary information

Supplementary Information

## Data Availability

The data that support the findings of the current study can be available from the corresponding author upon reasonable request.
