# Supplementary material for: Neurometabolic and functional connectivity basis of prosocial behavior in early adolescence
Source: Sci Rep. 2019 Jan 24;9:732. doi: 10.1038/s41598-018-38355-z (PMC6345858; doi:10.1038/s41598-018-38355-z)
Supplement: Supplementary file 1 — Supplementary Information [file 41598_2018_38355_MOESM1_ESM.docx]

**SUPPLEMENTARY INFORMATION**

**Neurometabolic and functional connectivity basis of prosocial behavior in early adolescence**

Naohiro Okada^1, 2^, Noriaki Yahata^1, 3^, Daisuke Koshiyama^1^, Kentaro Morita^1^, Kingo Sawada^1^, Sho Kanata^1, 4^, Shinya Fujikawa^1^, Noriko Sugimoto^1^, Rie Toriyama^1^, Mio Masaoka^1^, Shinsuke Koike^1, 2, 5^, Tsuyoshi Araki^1^, Yukiko Kano^6^, Kaori Endo^7^, Syudo Yamasaki^7^, Shuntaro Ando^1, 7^, Atsushi Nishida^7^, Mariko Hiraiwa-Hasegawa^8^, Richard A.E. Edden^9, 10^, Peter B. Barker^9, 10^, Akira Sawa^11^, Kiyoto Kasai^1, 2^

^1^ Department of Neuropsychiatry, Graduate School of Medicine, The University of Tokyo, Tokyo, Japan

^2^ International Research Center for Neurointelligence (WPI-IRCN), The University of Tokyo Institutes for Advanced Study (UTIAS), The University of Tokyo, Tokyo, Japan

^3^ Department of Molecular Imaging and Theranostics, National Institute of Radiological Sciences, National Institutes for Quantum and Radiological Science and Technology, Chiba, Japan

^4^ Department of Psychiatry, Teikyo University School of Medicine, Tokyo, Japan

^5^ The University of Tokyo Institute for Diversity and Adaptation of Human Mind (UTIDAHM), The University of Tokyo, Tokyo, Japan

^6^ Department of Child Psychiatry, Graduate School of Medicine, The University of Tokyo, Tokyo, Japan

^7^ Department of Psychiatry and Behavioral Sciences, Tokyo Metropolitan Institute of Medical Science, Tokyo, Japan

^8^ Department of Evolutionary Studies of Biosystems, School of Advanced Sciences, The Graduate University for Advanced Studies (SOKENDAI), Kanagawa, Japan

^9^ Russell H. Morgan Department of Radiology and Radiological Science, The Johns Hopkins University School of Medicine, Baltimore, MD, USA

^10^ F. M. Kirby Center for Functional Brain Imaging, Kennedy Krieger Institute, Baltimore, MD, USA

^11^ Department of Psychiatry, The Johns Hopkins University School of Medicine, Baltimore, MD, USA

**Table of Contents**

Supplementary Results ……………………………………………...……..4

Supplementary Methods ……………………………………………...……..6

Supplementary Figures ……………………………………………...……..13

**Supplementary Results**

**Associations between PB and psychological difficulties**

To deepen our understanding of the effect of PB on psychological difficulties in early adolescence, we investigated associations between SDQ PB scores and SDQ TD scores (data for one participant were missing). We observed a significant negative correlation between SDQ PB scores and SDQ TD scores (*ρ* = -0.36, *p* = 1.3×10^-9^), as illustrated in **Supplementary Fig. 1**.

**Associations between GABA and other metabolites**

To further elucidate neurometabolic interactions in early adolescence, we investigated associations between GABA and Glx concentrations and between GABA and tNAA concentrations. We observed a significant positive correlation between GABA and Glx concentrations (*ρ* = 0.16, *p* = 0.021) (**Supplementary Fig. 2a**), and a significant negative correlation between GABA and tNAA concentrations (*ρ* = -0.20, *p* = 3.6×10^-3^) (**Supplementary Fig. 2b**).

**Sex differences in PB, GABA, and FC**

We sought to explore sex differences in prosociality, GABA levels, and FC within the current sample. We examined sex differences in SDQ PB scales in all participants (data for one participant were missing). There were no significant sex differences in SDQ PB scales (*p* = 0.14) (**Supplementary Fig. 3a**)*.* Subsequently, we investigated sex differences in GABA levels. We observed no significant sex differences in GABA concentrations (*p* = 0.31) (**Supplementary Fig. 3b**). We also examined sex differences in seed-based FC (seeded in the left and right ACC), and found no brain region in which FC with the ACC was significantly different by sex.

**Supplementary Methods**

**Supplementary Method 1** Participants

The current study was performed as part of the population-neuroscience study of the TTC (pn-TTC) study, in which 301 early adolescents participated and biological markers including MRI, DNA methylations, and stress and gonadal hormones were measured. Participants of the pn-TTC study were subsampled from a larger participant group of the TTC study. The latter is a large-scale longitudinal population-based cohort survey in the Tokyo metropolitan area, in which 3,171 early adolescents have participated. Among participants in the TTC survey, those who showed an interest in the pn-TTC study were regarded as candidate participants. The included participants enrolled in the pn-TTC study approximately one year after participation in the TTC study. To check for any non-negligible sampling bias in the pn-TTC subsample, we compared basic attributes acquired in the TTC study between the participants and the non-participants in the pn-TTC study. We found no significant differences in age, sex, socioeconomic status, and intelligence quotient (*p* > 0.20). We confirmed that the pn-TTC subsample was representative of the original TTC study population. Written informed consent was obtained from each participant and the participant’s primary parent before participation. All protocols were approved by the research ethics committees of the Graduate School of Medicine and Faculty of Medicine at the University of Tokyo (approval no. 3150, 10057, and 10069), Tokyo Metropolitan Institute of Medical Science (approval no. 12–35), and the Graduate University for Advanced Studies (SOKENDAI) (approval no. 2012002). All research was performed in accordance with relevant guidelines/regulations. During their first visit, participants were introduced to a mock scanner to acclimatize to the MRI scanner environment, and to practice lying still during the scan. During their second visit, participants underwent MRI scanning. The exclusion criteria for participation included: (a) evident psychiatric or neurological disorder (e.g., ASD, ADHD, Down syndrome, or epilepsy); (b) visual or auditory impairment (except myopia); (c) endocrinological disease, or disease that might have an effect on the hypothalamic-pituitary-adrenal axis function (e.g., diabetes mellitus, thyroid disease, or renal dysfunction), gonadotropic dysfunction, or adrenal dysfunction; (d) recent or long-term use of drugs that might influence the central nervous system (e.g., steroid hormones and antihistamines); (e) history of head trauma with loss of consciousness for five minutes or more; (f) metal implants (except titanium) in the body; (g) unrest during the MRI practice session in the first visit.

**Supplementary Method 2** Psychological evaluation

The SDQ is a common parent-rated instrument for assessing psychopathology in children. The SDQ includes five factors: emotional symptoms, conduct problems, hyperactivity/inattention, peer problems and PB. Scores for each SDQ factor range from 0 to 10. The higher the SDQ factor score is, the greater the child’s difficulty is, except for the PB factor. The TD score is calculated by summing four problem scales (emotional symptoms, conduct problems, hyperactivity/inattention, and peer problems, that is, all except for the PB scale). Participants had already completed the SDQ at the time of participation in the TTC study. The mean interval between psychological assessment with the SDQ and MRI scanning was 16 months (ranging from 7 to 39 months). In addition to the PB scores, TD scores were used as a reference variable in the current study.

**Supplementary Method 3** Subject selection

Of the 301 participants enrolled in the pn-TTC study, a total of 271 early adolescents (129 girls and 142 boys) with a mean age of 11.5 years, underwent the designed series of MRS scanning and/or rsfMRI scanning. All 271 participants also underwent FLAIR and MRA scanning to rule out abnormal organic findings. Furthermore, 253 early adolescents (119 girls and 134 boys) underwent the above-mentioned MRS examination. After excluding data with abnormal organic findings and/or low spectral quality, 221 early adolescents (106 girls and 115 boys) with a mean age of 11.5 years were used for the subsequent GABA analyses. There were 257 early adolescents (121 girls and 136 boys) who underwent the above-mentioned rsfMRI scanning. After excluding data with abnormal organic findings and/or low data quality, 187 early adolescents (92 girls and 95 boys) with a mean age of 11.6 years were used for the subsequent rsfMRI analyses. Finally, 171 early adolescents (83 girls and 88 boys) with a mean age of 11.6 years were used for the analyses combining both rsfMRI and MRS.

Therefore, we had four neuroimaging samples: all participants, the GABA-MRS analysis sample, the rsfMRI analysis sample, and the combination analysis sample.

**Supplementary Method 4** Supplementary analyses

First, to deepen our understanding of the effect of PB on psychological difficulties in early adolescence, we investigated associations between SDQ PB scores and SDQ TD scores with Spearman’s rank tests. Second, to deepen our understanding of neurometabolic interactions in early adolescence, we investigated associations between GABA and Glx concentrations and between GABA and tNAA concentrations with Spearman’s rank tests. Third, we sought to explore sex differences in prosociality, GABA levels, and FC within the current cohort sample. We examined sex differences in SDQ PB scales and GABA levels with Mann-Whitney U tests, and sex differences in seed-based FC (seeded in the left and right ACC) with second-level linear regression analyses in SPM12.

**Supplementary Figures**


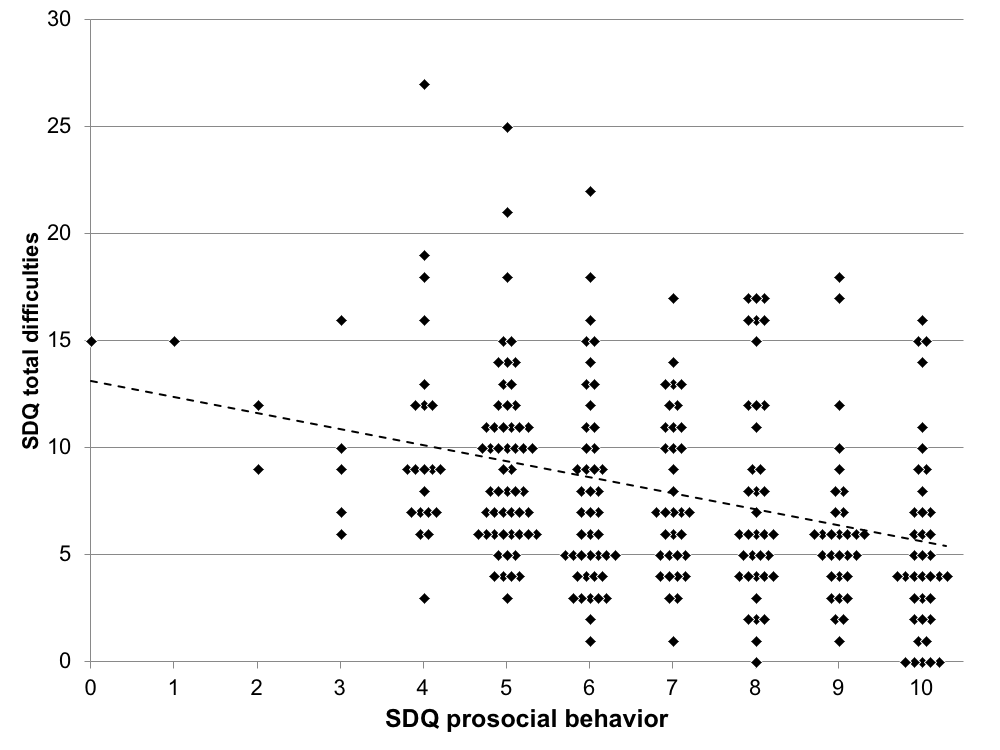


**Supplementary Figure 1** Associations between the Strengths and Difficulties Questionnaire (SDQ) prosocial behavior (PB) scores and SDQ total difficulties (TD) scores (data for one participant missing)

Multiple points that are originally on the same coordinate are shown separately for display purpose.


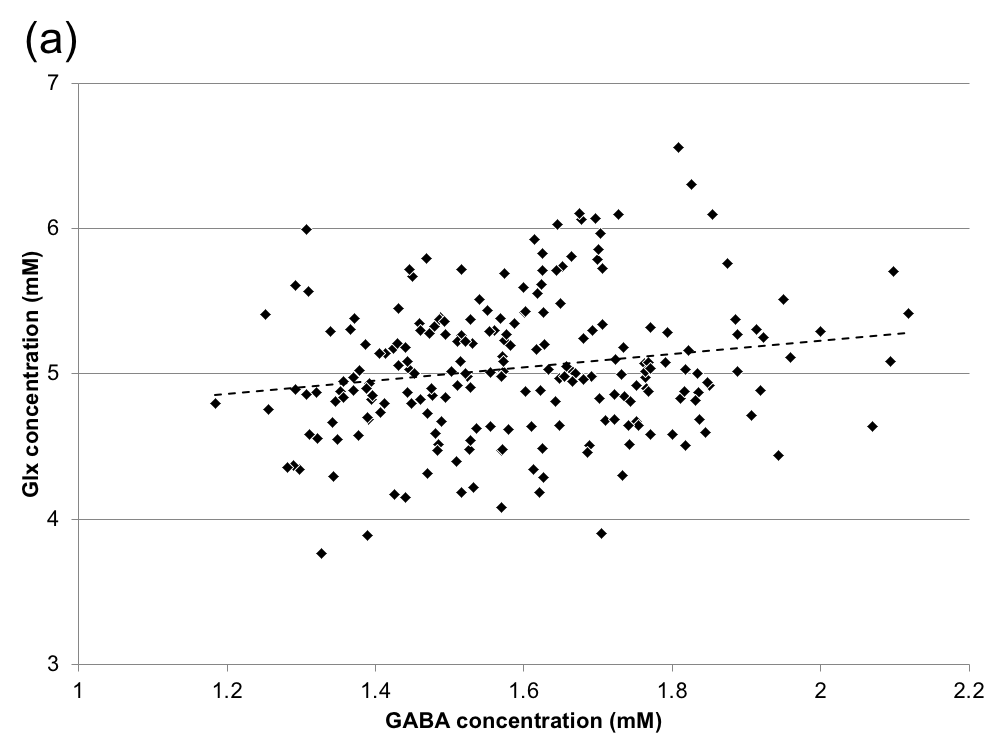

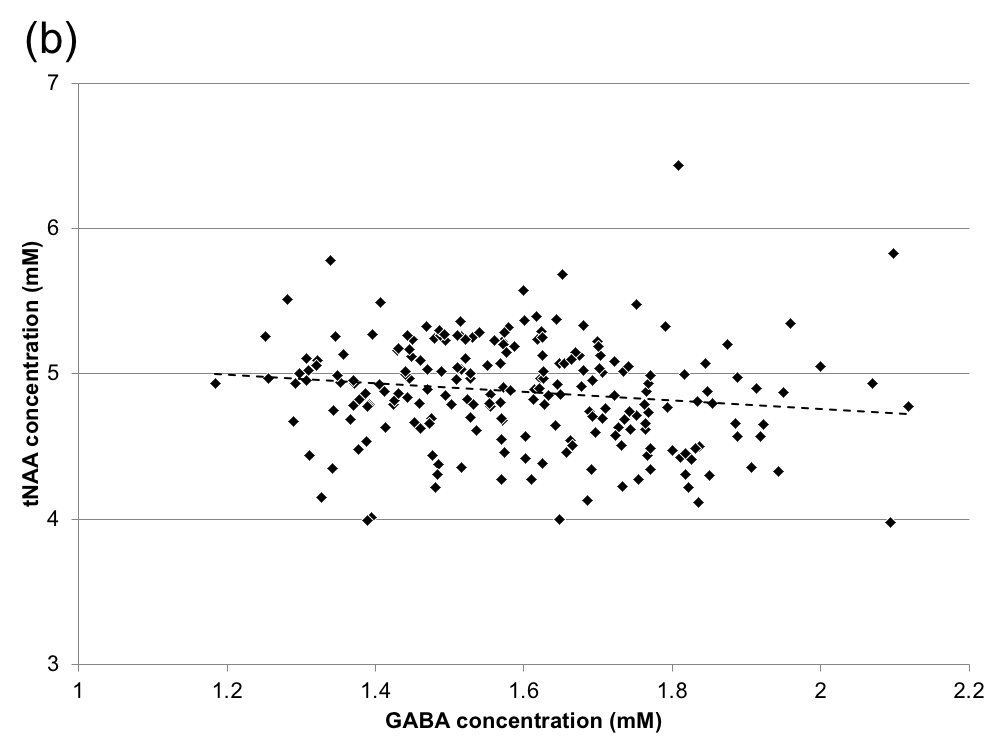


**Supplementary Figure 2** Intercorrelations of neurometabolite concentrations

**(a)** Correlation between GABA and Glx (the sum of glutamate and glutamine) concentrations in the anterior cingulate cortex (ACC). **(b)** Correlation between GABA and tNAA (the sum of NAA and NAAG) concentrations in the ACC.


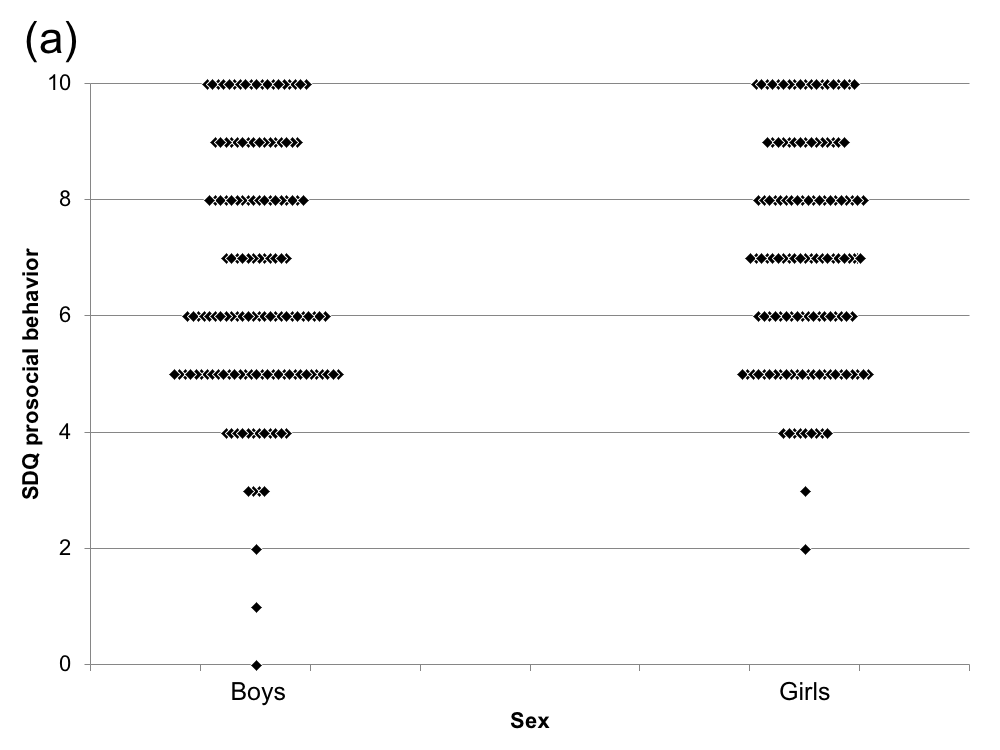

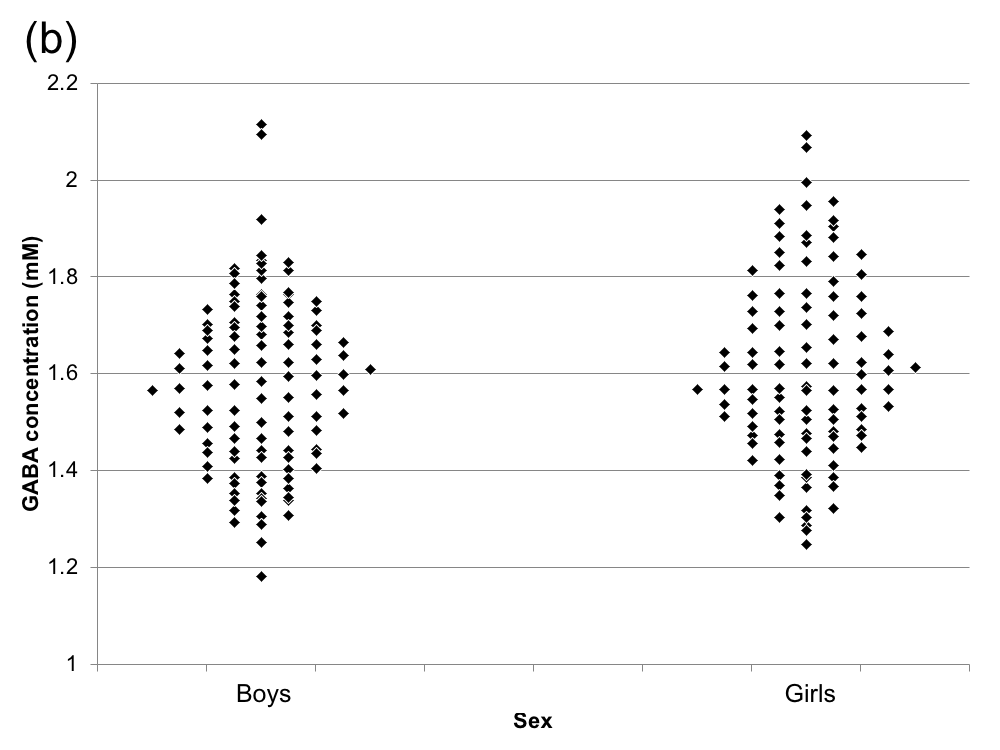


**Supplementary Figure 3** Sex differences

**(a)** Sex differences in the Strengths and Difficulties Questionnaire (SDQ) prosocial behavior (PB) scores in all participants (data for one participant missing). **(b)** Sex differences in GABA concentrations in the anterior cingulate cortex (ACC).
